# Tuberculosis Monitoring Encouragement Adherence Drive (TMEAD): Toward improving the adherence of the patients with drug-sensitive tuberculosis in Nashik, Maharashtra

**DOI:** 10.3389/fpubh.2022.1021427

**Published:** 2022-12-21

**Authors:** Somen Saha, Deepak Saxena, Devang Raval, Nishad Halkarni, Rahul Doshi, Madhav Joshi, Mridhula Sridharan, Jignasa Sathwara, Sandul Yasobant, Harsh Shah, Zahiruddin Syed Quazi, Kavitha Rajsekar, Jayeeta Chowdhury

**Affiliations:** ^1^Department of Public Health Science, Indian Institute of Public Health Gandhinagar (IIPHG), Gandhinagar, Gujarat, India; ^2^School of Epidemiology and Public Health, Datta Meghe Institute of Medical Sciences (DMIMS), Wardha, Maharashtra, India; ^3^Sensedose Technologies, Nashik, India; ^4^India Health Fund, Mumbai, India; ^5^Department of Health Research, Ministry of Health and Family Welfare, Government of India, New Delhi, India

**Keywords:** tuberculosis, adherence, India, digital adherence technology, National TB programs, incremental cost-effectiveness ratio (ICER)

## Abstract

**Introduction:**

Adherence to tuberculosis (TB) medication is one of the critical challenges to tuberculosis elimination in India. Digital adherence technologies (DAT) have the potential to facilitate medication adherence and monitor it remotely. Tuberculosis Monitoring Encouragement Adherence Drive (TMEAD) is one such DAT piloted in Nasik, Maharashtra, from April 2020 to December 2021. The study aims to assess the adherence and cost-effectiveness of TMEAD compared to the standard of care among patients with drug-sensitive tuberculosis (DSTB) residing in the urban areas of Nasik, Maharashtra, India.

**Methods:**

A quasi-experimental study was conducted among new cases of TB as per the National TB Elimination Programme (NTEP) residing in the urban geography of Nasik. The intervention and control arms were purposively selected from non-contaminating TB units (TUs). A total of 400 DSTB patients (200 in the intervention group and 200 in the control group) were enrolled. After enrolment, patients in the intervention arm were provided with the TMEAD device and followed for 24 weeks to assess treatment outcomes. Adherence was measured as those patients who have completed 80% of prescribed doses, as reported during patient follow-up, and further validated by analyzing the trace of rifampicin in urine among 20% of patients from both arms. A budget impact analysis was done to assess the impact of the TMEAD program on the overall state health budget.

**Results:**

Out of 400 enrolled DSTB patients, 261 patients completed treatment, 108 patients were on treatment, 15 patients died, and 16 patients were defaulters over the study period. The study reported overall treatment adherence of 94% among those who completed treatment. Patient reports indicated high levels of treatment adherence in the intervention group (99%) as compared to the control group (90%). Adherence assessed through analyzing trace of rifampicin in the urine sample for the intervention arm was 84% compared to the control arm (80%). Per beneficiary (discounted) cost for TMEAD was Indian rupees (INR) 6,573 (USD 83). The incremental cost-effectiveness ratio of the intervention is INR 11,599 (USD 146), which shows that the intervention is highly cost-effective.

**Conclusion:**

This study revealed that patient-reported treatment adherence was high in TMEAD when compared to standard therapy of care for DSTB patients and the intervention is cost-effective. TMEAD could complement the national strategy to end TB by improving adherence to the treatment regimen in India.

## Introduction

India aims to end tuberculosis (TB) by 2025, 10 years before the sustainable development goals (SDG) target, with the strategic implementation of the National Tuberculosis Elimination Program (NTEP) (1). Although TB can be cured with a first-line anti-TB antibiotic treatment regimen, non-adherence is the main challenge for TB control and prevention programs (2–4). As per a systematic review, pooled prevalence estimates of drug resistance TB (DR TB) and multi-drug resistance TB (MDR TB) from 2006 to 2015 were the highest in Western India (5). Treatment adherence is challenging, given the complexity, modest tolerability, and long duration of the treatment regimens currently available for both drug-susceptible and drug-resistant TB. In turn, low adherence increases the risk of poor outcomes, including treatment failure, relapse, and the development or amplification of drug resistance (6).

The expansion of mobile phones and cellular access, digital adherence technologies (DATs), is facilitating alternative approaches for improving adherence. These technologies range from cell phone short messaging service (SMS) text to digital pillboxes, to ingestible sensors. DATs use cellular communication and other innovations to perform a variety of functions, including reminding patients to take medications, digitally observing doses taken, and compiling dosing histories that can be used by healthcare providers (HCPs) to identify and intervene in non-adherence (7, 8).

Various DATs have been adopted in India, including the 99DOTS (Direct Observed Treatment Short Course) system to monitor patients' compliance with TB treatment. In 99DOTS, the patients are provided with an anti-TB blister pack wrapped in a custom envelope, including hidden phone numbers visible only when doses are dispensed (4). After taking their daily medication, patients make a free call to the hidden phone number, indicating that the dose has been taken. The 99DOTS patients received a series of daily reminders *via* SMS and automated calls. Missed doses trigger SMS notifications to care providers, who follow up with personal, phone-based counseling. Real-time adherence reports were also available on the web (9, 10). Among others, the Medication Event Reminder Monitor (MERM) is a digital pillbox that provides pill-taking reminders and facilitates remote medication adherence monitoring (11).

### Tuberculosis Monitoring Encouragement Adherence Drive (TMEAD)

**Figure 1:**
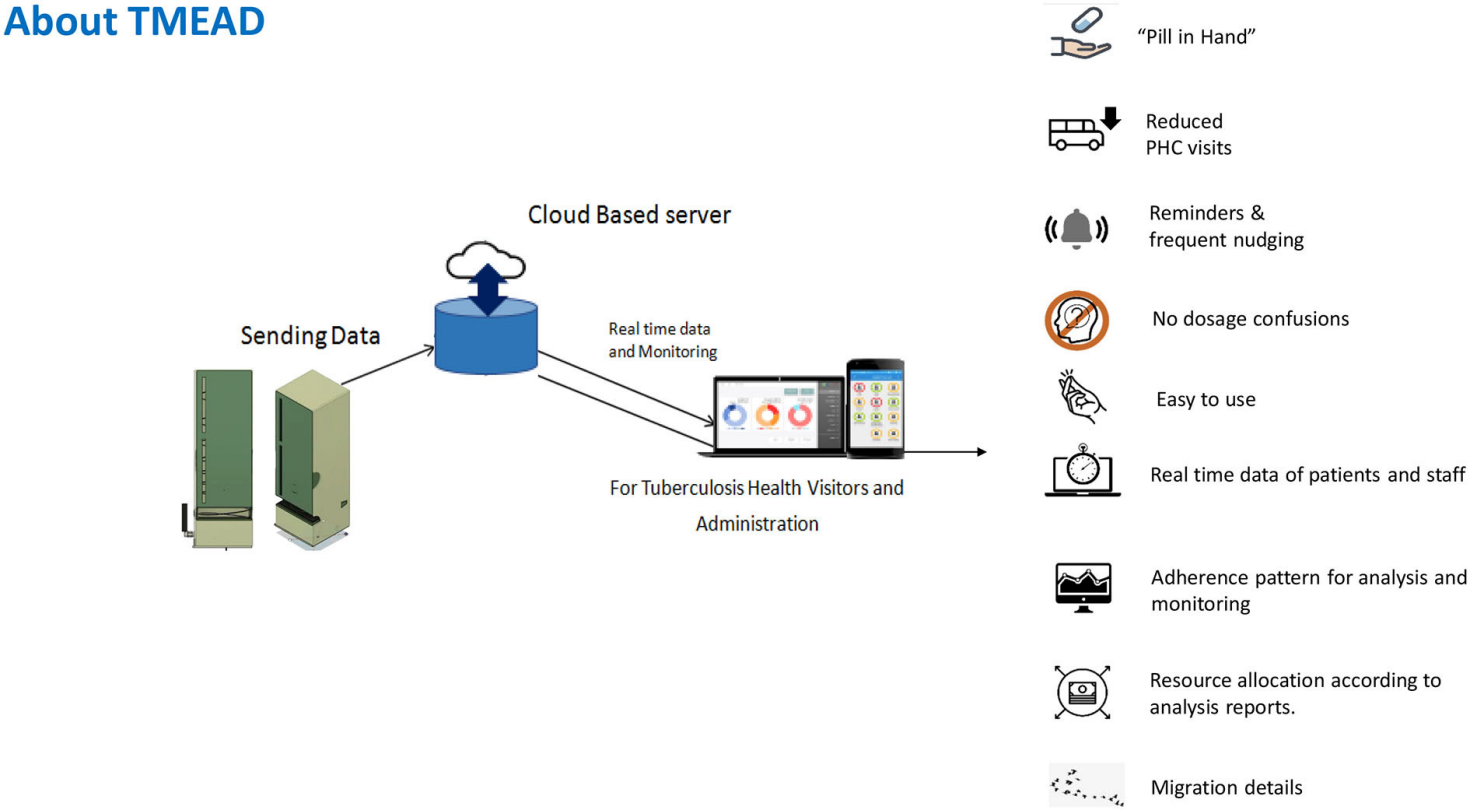
Overview of the solution.

Tuberculosis Monitoring Encouragement Adherence Drive–a new form of digital adherence technology–was piloted among newly diagnosed drug-sensitive pulmonary TB (DSTB) cases in Maharashtra. TMEAD helps monitor and ensure patient compliance. A physical device equipped with an integrated software-linked mobile network reminds, dispenses, and senses a patient's adherence to the treatment regime. A web-based application provided real-time monitoring with daily updates and patient analytics to peripheral health institutions (PHI). A mobile application provides instant updates and a quick view for the TB Health Visitor (TBHV) when they are on the field. It also creates a detailed, automated adherence dashboard of all patients with TMEAD for health workers and policymakers to prioritize their resources toward patient adherence^1^. TMEAD is a potential solution for both the patients and the program staff. Figure 1 shows an overview of the TMEAD solution.

## Aim and objectives

The study aims to assess the adherence to medications and cost-effectiveness of TMEAD compared to the standard of care for patients with DSTB residing in the urban geography of Nasik, Maharashtra, India. The study's primary objective is to measure treatment adherence with TMEAD compared to the usual care scenario.

## Methods

### Study settings

A quasi-experimental study was conducted among new cases of TB as per NTEP residing in the urban geography of Nasik. The intervention and control arms were purposively selected from non-contaminating TB units (TUs). To implement NTEP, each district has a district TB center, which monitors the program for the entire district. The district is further divided into sub-districts, i.e., a tuberculosis unit (TU) at each block. Urban Nasik, considered a district unit, is spread across five TUs. The TUs were assigned into two arms, ensuring that they are geographically apart, thereby reducing the possibility of contamination. The participants were then divided into two arms: intervention and control. Ethical permission for the study was obtained from the Institutional Ethics Committee of the Indian Institute of Public Health in Gandhinagar. Administrative approval from the health department and the Nasik City TB office was also taken.

### Study design

The primary study comprised a longitudinal follow-up of the patients assigned to the intervention arm with TMEAD and the comparison arm with the usual care scenario. Based on an assumption of an increase in the adherence to TB treatment from 80 to 95% (as desired under NTEP guideline) with 95% confidence and 80% power and assuming a 20% of dropout/non-response/attrition, the proposed sample size in each of the arm was 200. The quantitative survey included the collection of patients' demographic details from the selected TUs and ensured the baseline matching of the study participants in both arms. Once the patients were enrolled in the intervention arm, the TMEAD device was provided. The trained research team engaged with patients and household members in explaining the mechanism of functioning of the box and also explained the finer details such as when to charge, how to remove the tablet box from the TMEAD device, and what to do if the medicine pill packets have not been removed. The patients in the intervention arm were also informed about the follow-up protocol. The patients enrolled in the control arm were also followed up as per the NTEP guidelines. As per the protocol, the study participants were followed longitudinally for 24 weeks from the start of their treatment to assess the outcome of treatment. The patients' treatment outcomes were categorized into successful treatment, defaulters, and death.

### Adherence measurement

Treatment completion for DS-TB was recorded on the refill dispensing of a minimum of 168 days of drugs within 240 days (8 months) from the treatment initiation date, without evidence of a treatment interruption of 1 month or more. WHO defines “adherence to medication” as the extent to which patients take their medications as prescribed with respect to dosage and intervals throughout the treatment period (12). In general, adherence was defined as the extent to which the patient's prescribed dosing regimen is followed, where the denominator comprises the number of days into treatment (from the treatment initiation date) and the numerator includes the number of days for which the prescribed number of doses are taken. In this study, the level of adherence was defined as the number of patients who had completed 80% of the prescribed doses for treatment completion. For those patients who were on treatment after the study duration but had completed more than 8 weeks of treatment, point adherence was calculated.

Furthermore, adherence was also assessed by analyzing rifampicin traces in urine among 20% of patients enrolled from both arms. The presence of rifampicin was considered a confirmation of the dose taken in the given time period. The high-performance liquid chromatography (HPLC) method was used for urine analysis among patients with DSTB. The first sample of urine was collected during the intensive phase, the second sample within 1 month, and the third sample within 1 month of the second sample. Results were reported for sample positivity in any round. A cutoff value of 100 μg was considered for reporting 24 h of adherence to medication. Chi-square tests were performed to check the statistical significance between the intervention and control groups.

Patients were enrolled in the study within 2 weeks of enrolment in the DSTB regimen. The research team followed up with the patients weekly to document their experience with treatment. Information on factors that influence TB treatment regimen adherence and a baseline score of socioeconomic variables (e.g., age, gender, housing type, and asset ownership to assess income level, occupation, and education level) were compared to drug compliance level (including distance to the clinic and level of support from family members).

A Tuberculosis Monitoring Encouragement Adherence Drive device was deployed for DS-TB patients in April 2020. The patient was enrolled in the study until the sample size was achieved. The patient was followed up until 31 December 2021, and their outcomes were collected. During each of the follow-ups, adherence was assessed.

### Valuing of health outcomes

Health-related quality of life (HRQoL) was assessed using the EQ-5D-5L tool at baseline and the first follow-up. The tool has five domains: mobility, self-care, usual activities, pain/discomfort, and anxiety/depression. Given a score range from 1 to 5, with one being the worst and five the best (13), the level of problem reported on each of the EQ-5D dimensions determines a unique health state. Health states were converted into a weighted health state index by applying scores from the EQ-5D preference weights elicited from general population samples using the Crosswalk Index calculator (14–16). These weights lie on a scale on which full health has a value of 1 and dead a value of 0. For this study, Thailand population weights were converted to an EQ-5D index score.

## Measuring the cost of care

Assumption (based on the field practical experiences): each device can be reused twice. The cost related to TMEAD devices was obtained from the implementing partner. The cost was calculated under the three costing heads, namely, manufacturing costs, variable costs, and human resource costs. The cost related to the standard of care was obtained from the secondary literature by adjusting the mean of the costs with the gross domestic product (GDP) deflator rate (Table 1) (17–19). As per WHO guidelines, all future costs and consequences were discounted by 3%. All costs are reported in INR and USD, with 1 USD ~ 79.58 INR for the year 2020.

**Table 1:** Standard care (control arm) cost for DSTB patients.

**S. No**	**GDP for the referenced year**		**GDP for the** **study year 2020**	**Cost of** **care (INR)**	**Cost of constant** **price (INR)^#^**	**References**
1	2005	61.4	146.1	1,398	3,326	(17)
2	2006	52.9	146.1	3,024	8,352	(18)
3	2020	139.7	146.1	2,500	2,614	(19)
The average cost in the control arm					4,764	

# Cost in the constant price calculated as the cost of care mentioned in the referenced article multiplied by GDP for the study year 2020 divided by GDP for the referenced year.

### A conceptual framework for a decision tree model

A decision tree was parameterized on an MS Excel spreadsheet to estimate the change in quality-adjusted life years (QALY) and cost from a societal perspective. In the decision tree model, the intervention (TMEAD) was compared to the control arm (standard of care). The adherence was categorized as follows:

- Full adherence (patients achieving above 90% of adherence)
- Partial adherence (between 80 and 90%)
- Non-adherence (below 80% adherence).

Treatment outcomes like treatment completed, treatment extended, death, and defaulter were modeled to estimate QALY gained. For modeling purposes, we have excluded patients who were on treatment.

**Figure 2:**
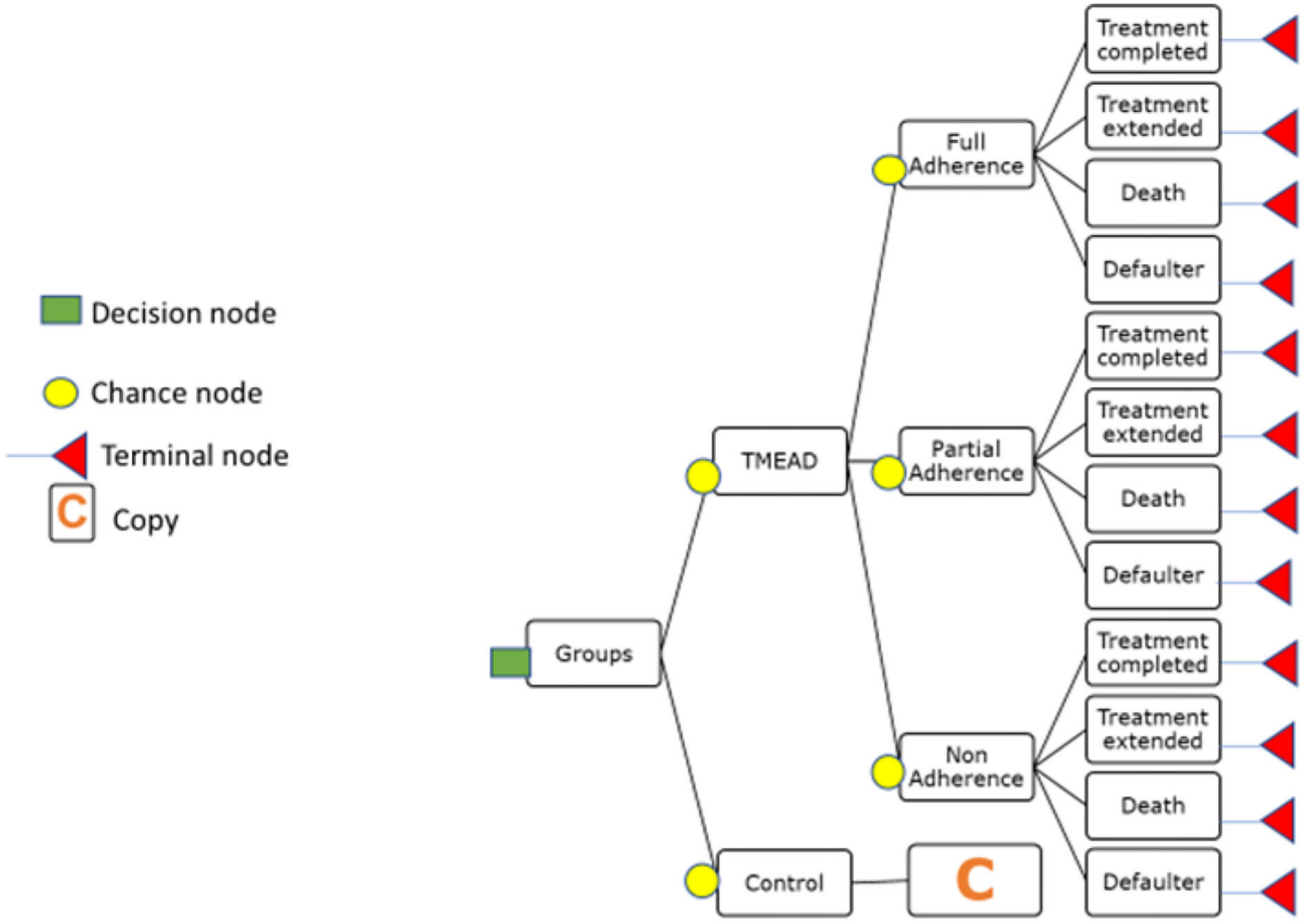
Decision tree model.

Transition probability was derived from primary as well as secondary literature. The transition probability of the TMEAD and standard of care was calculated based on the proportional distribution of the patients under each adherence category. The probability of the TMEAD device for full adherence for the treatment-completed group, for the treatment-extended group, death, and defaulter were calculated from the proportional distribution of the patients under each outcome. Likewise, the probability of the TMEAD device for partial adherence and non-adherence was calculated from the proportional distribution of the patients with respect to each treatment outcome. The transition probability of QALYs was calculated using the EQ-5D utility value (Thailand index) for the full adherence, partial adherence, and non-adherence groups. The treatment cost of the TMEAD device was calculated from the per-beneficiary cost of the TMEAD device. Furthermore, the cost of treatment by the TMEAD device was apportioned by adherence category, i.e., full adherence, partial adherence, and non-adherence. The cost of full adherence was apportioned to treatment completed, treatment extended, death, and defaulter. Similarly, the cost of partial adherence and non-adherence were calculated with each treatment outcome. The cost of death and defaulters under full adherence and partial adherence was zero, as there were no deaths and defaulters in that group. The average age of the participants in both arms was reported from the primary data. The same calculations were applied to the control arm. A decision tree model was developed and prepared by using TreeAge Pro Software Healthcare Version 2022 R.1, as shown in Figure 2.

### Measuring the cost-effectiveness

The decision tree model calculated the total cost and total QALYs gained for the interventions and control. The incremental cost/QALY was the difference in the total cost/QALY between the intervention and the control. The incremental cost-effectiveness ratio (ICER) was obtained by taking the ratio of incremental cost and incremental QALY. The CEA outcomes were expressed in cost per QALY gained. The time horizon of the study was 1 year, and a 3% discount was applied. We applied gross domestic product (GDP) per capita based on the WHO guideline for willingness to pay threshold and considered an ICER of less than GDP per capita as highly cost-effective. In our study, India's 2020 GDP per capita of INR 1,45,679 (~USD:1830) has been considered the cost-effectiveness threshold value per QALY gained.

### Sensitivity analysis

The robustness of the model was assessed using one-way sensitivity analysis (OWSA). In one-way sensitivity analysis, 95% CI values for utility values and 25% upper/lower values for the other model input parameters were used and reported as tornado diagrams.

### Budget impact analysis (BIA)

A budget impact analysis (BIA) was performed to estimate the cost for the roll-out of TMEAD intervention in the state of Maharashtra. The BIA was performed at 2021 prices. The following assumptions were made in the BIA.

i) The annual economic model holds true for 5 years.
ii) The annual budget was based on the unit cost of manufacturing, capital cost, and HR cost assumed in the Health System Perspective CEA model for DSTB patients.
iii) The uptake of rolling out the device was taken between 30 and 65% for 5 years.

The Budget Impact Analysis depicts budget allocation for 5 years. Using a top-down approach, we calculated the eligible population, and supply side costing was used to assess incremental costs of intervention to be delivered in a horizontal platform.

### Statistical analysis

Descriptive statistics were used to describe the sample. For continuous variables, we reported the mean and range in each arm; to compare groups, considering a normal variable distribution, an independent sample *t*-test was applied. For categorical variables, we performed chi-square tests to study possible associations between groups. All statistical analyses were performed using the Statistical Package for Social Science (SPSS for Windows, version 20; SPSS, Chicago, IL) program. A *p*-value of < 0.05 was considered to be statistically significant.

## Results

This study was conducted in the urban area of the city of Nasik, where a total of 400 patients were enrolled. The patients were allocated to two arms, namely, the intervention arm and the control arm. Efforts were made to include TUs in the respective arm, which was non-contaminating.

### Sociodemographic profile

The sociodemographic characteristics of the patients in the intervention and control arms are presented in Table 2. The study had 43.8% of male participants and 56.3% of female participants. The mean age of the patients was 37 (SD ± 14) years, ranging from 18 to 92 years. It was observed that around one-third of the participants were from the general category, 34.5 and 33.5% in the intervention and control arms, respectively.

**Table 2:** Baseline sociodemographic characteristics of the study participants.

**Variables**	**Intervention** **(%)**	**Control** **(%)**	**Total** **(%)**	***p*** **-value**
**Total number of participants**	**200**	**200**	**400**	
**Gender and age**				
Male	88 (44)	87 (43.5)	175 (43.8)	0.920
Female	112 (56)	113 (56.5)	225 (56.3)	
Age in mean years	37 [18–92]	37 [18–92]	37 [18–92]	0.543
**Caste**				
General	69 (34.5)	67 (33.5)	136 (34)	0.018
S.C/S. T	79 (39.5)	57 (28.5)	136 (34)	
Other Backward Caste	52 (26)	76 (38)	128 (32)	
**Religion**				
Hindu	182 (91)	175 (87.5)	357 (89.3)	0.060
Muslim	15 (7.5)	25 (12.5)	40 (10)	
Others	3 (1.5)	0 (0)	3 (0.8)	
**Education**				
Literate	60 (30)	90 (45)	150 (37.5)	0.002
Illiterate	140 (70)	110 (55)	250 (62.5)	
**Marital status**				
Unmarried	49 (24.5)	51 (25.5)	100 (25)	0.154
Married	135 (67.5)	142 (71)	277 (69.3)	
Divorced/ widowed/ separated	16 (8)	7 (3.5)	23 (5.8)	
**Occupation**				
Unemployed	81 (40.5)	108 (54)	189 (47.3)	0.004
Private employee	49 (24.5)	55 (27.5)	104 (26)	
Government employee	8 (4)	2 (1)	10 (2.5)	
Laborer	59 (29.5)	33 (16.5)	92 (23)	
Self employed	3 (1.5)	2 (1)	5 (1.3)	
**General profile**				
Below poverty line population	72 (36)	65 (32.5)	137 (34.3)	0.164
Monthly expenditures in HH	2,010 [500–8,000]	2,628 [0–25,000]	2,414 [0–25,000]	0.010

[ ] Indicates range.

( ) Indicates percentage.

SC, scheduled caste; ST, scheduled tribe; HH, household.

The majority of the patients (89.3%) were Hindu; 91 and 87.5% were in the intervention and control arms, respectively. Overall, the literacy level was low (37.5%), about 70% in the intervention arm and 55% in the control arm were illiterate. It was observed that 47.3% of the patients were unemployed, compared to 40.5 and 54% in the intervention and control arms, respectively. A total of one-third of the population was BPL, which included 36 and 32.5% in the intervention and control arms, respectively. The average monthly expenditure in the household was INR 2,414 (USD: 30), INR 2,010 (USD: 26), and INR 2,628 (USD: 33) in the intervention and control arms, respectively. Statistically significant differences were found between the intervention and control groups regarding education, caste, occupation, and monthly household expenditure.

### Treatment history

Among the patients included in the present study, in the intervention arm, 41.5% were detected with TB in 2020 and the rest in 2021. Overall, 61% of the patients in the intervention arm were detected with TB in government hospitals, whereas only 49% in the control arm were detected with TB in the government setup. The median delay of 23 days from the onset of the symptoms to diagnosis was greater in the intervention arm (28 days) compared to the control arm (20 days). About 47% of the study population had a delay between diagnosis and treatment. The reason might be due to migration, lack of acceptance of TB treatment, lack of awareness, COVID-19 challenges, and poor health-seeking behavior. The majority (89.3%) of the study patients had chosen government facilities because of the free treatment, which was affordable and convenient. Table 3 describes the treatment history of the study population. All differences between the intervention and control groups were statistically insignificant.

**Table 3:** Treatment history.

**Variables**	**Intervention (%)**	**Control (%)**	**Total (%)**	***p*** **-value**
**Total number of participants**	**200**	**200**	**400**	
**Year of TB detection**				
2020	83 (41.5)	87 (43.5)	170 (42.5)	0.686
2021	117 (58.5)	113 (56.5)	230 (57.5)	
**First place of diagnosis**				
Government (Public)	122 (61)	98 (49)	220 (55)	0.016
Private	78 (39)	102 (51)	180 (45)	
**Duration between onset of the symptoms to diagnosis (in days)**	28 [0–60]	20 [3–73]	23 [0–73]	
**Reasons for the delay between diagnosis and treatment**				
No delay	124 (62)	87 (43.5)	211 (52.8)	0.456
Did not have time to go to providers	9 (4.5)	5 (2.5)	14 (3.5)	
Others (specify)				
Migration				
Lack of acceptance				
Lack of awareness/no answer	67 (33.5)	107 (53.5)	174 (43.5)	
Lockdown and COVID-19 challenges				
Poor health seeking behavior				
**Travel**				
Mean distance (KMs)	5 [0–17]	5 [2–25]	6 km [0–25]	0.052
Mean time (Min)	16 [0–66]	25 [5–60]	21 [0–66]	0.056

( ) Indicates percentage.

[ ] Indicates range.

## Adherence

Adherence to treatment was calculated after the third follow-up. Adherence is defined as the extent to which the patient's prescribed dosing regimen is followed. The denominator is the number of days in treatment since the treatment initiation. The numerator is the number of days for which the prescribed number of doses were taken. Overall adherence was 94% among those who completed treatment (Table 4). Adherence in the intervention arm was 99%, compared to 90% in the control arm. Point adherence among those who are on treatment was 97.4%, with higher adherence reported in the intervention arm (98.7%) compared to 95.2% in the control arm. The differences in adherence among both patients who completed treatment and those who were on treatment were statistically significant.

**Table 4:** Treatment outcome and adherence.

**Treatment outcomes and adherence**	**Intervention (%)**	**Control (%)**	**Overall (%)**	***p*** **-value**
**Total number of participants**	**200**	**200**	**400**	
Treatment completed	122 (61)	139 (69)	261 (65)	0.150
Default/failure	6 (3)	10 (5)	16 (4)	
Died	6 (3)	9 (4.5)	15 (3.6)	
On treatment	66 (33)	42 (21)	108 (27)	
Treatment completed_adherence	99 [98.3–99.7]	90 [89.3–90.7]	94 [93.2–94.7]	0.012
On treatment_point adherence	98.7 [97.9–99.45]	95.2 [94.5–95.9]	97.4 [96.6–98.1]	0.018

( ) Indicates percentage.

[ ] Indicates confidence interval.

### Urine rifampicin analysis

A 24-h recall of drug consumption was elicited, and those who had consumed the tablets were requested to give their urine samples. A total of 104 samples were collected in the intervention arm, which included 40, 38, and 26 over three cycles, respectively. However, in the control arm, 108 samples were collected which included 40, 38, and 30 over three cycles, respectively. The number of samples collected was more or less similar. However, the number of samples processed in the intervention arm was 36, 32, and 22, respectively. While in the control arm, the number of samples processed was 37, 31, and 22, respectively.

It was observed that in the intervention arm out of the total samples processed in the first cycle, 91% had urine rifampicin traces. At the end of the third cycle of urine sample collection in the intervention arm, 86.3% of samples were positive for urine rifampicin. While in the control arm, 77.3% of the samples were rifampicin positive (Table 5).

**Table 5:** Urine analysis across all rounds.

**Group**	**Presence of rifampicin in Urine**					
	**1st round**		**2nd round**		**3rd round**	
	***N***	**%**	***N***	**%**	***N***	**%**
Intervention	29	91 [90.2–91.0]	28	88 [87.3–88.3]	19	86.3 [85.7–86.9]
Control	30	81 [80.7–81.3]	25	80.6 [80.4–80.6]	17	77.3 [76.7–77.9]

[ ] Indicates confidence interval.

### Health-related quality of life (HQoL)

Health-related quality of life of patients was assessed using the EQ5D5L tool. We used EuroQol's Crosswalk value sets for Thailand using the EQ5D5L profile. The EQ5D index score of DSTB patients in the intervention arm is 0.62 and 0.64 in the control arm, which is close to being completely healthy and indicates that the treatment of TB significantly affected the HRQOL of the patients (Table 6).

**Table 6:** EQ5D5L index values of study participants.

	**Intervention**	**Control**
EQ5D5L profile	12,212	21,113
Index score	0.626	0.666

## Cost of TMEAD

The total annualized cost of the program implementation for the intervention was INR 13,55,324. Table 7 shows a summary of key costs and per beneficiary costs. The intervention is INR 6,573. The per beneficiary cost for the standard of care was INR 4,764.

**Table 7:** Per beneficiary cost of the intervention (TMEAD device).

**Sr. No**	**Particulars**	**Total program** **cost (INR)**	**Total program** **cost (USD)**	**Remarks**
1	Manufacturing cost	2,03,486.2	2,557.3	A total of 200 devices deployed.
2	Implementation cost	1,73,424.1	2,179.7	Server support, SIM cost, SMS service, training, transportation and AMC/repairs
3	HR costs	9,78,413.5	12,297.2	Cost of human resources included service engineer, app developer, web developer, electronics hardware engineer, program manager, helper, and operation manager
Total annualized cost		13,55,324	17,034.6	
Per beneficiary cost		6,573	82.6	Applying 3% discount

## Cost-effectiveness analysis

The total cost and total QALYs gained for the interventions and control were calculated from the decision tree model. The incremental cost/QALY was the difference in the total cost/QALY between the intervention and the control. ICER was obtained by taking the ratio of incremental cost and incremental QALY. We applied Gross Domestic Product (GDP) per capita based on the WHO guideline for willingness to pay threshold and considered an ICER of < 1 GDP per capita as highly cost-effective. In our study, India's 2020 GDP per capita of INR 1,45,679 (USD: 574) is considered the cost-effectiveness threshold value per QALY gained. TMEAD incurs an incremental cost of INR 11,599 (USD: 146) per QALY gained, which is 0.07% of the per capita GDP of India. This suggests our intervention is highly cost-effective as compared to the control. Table 8 shows the results of the cost-effectiveness analysis between intervention and control.

**Table 8:** Results of cost-effectiveness analysis between intervention and control.

**Outcomes**	**Intervention**	**Control**
Cost in INR (USD) per patient treated as per modeling	6,573 (83)	4,764 (60)
Difference in Cost in INR (USD)	2,042 (26)
Difference in QALYs	0.176
ICER	11,599 (146)

### Cost-effectiveness plane

**Figure 3:**
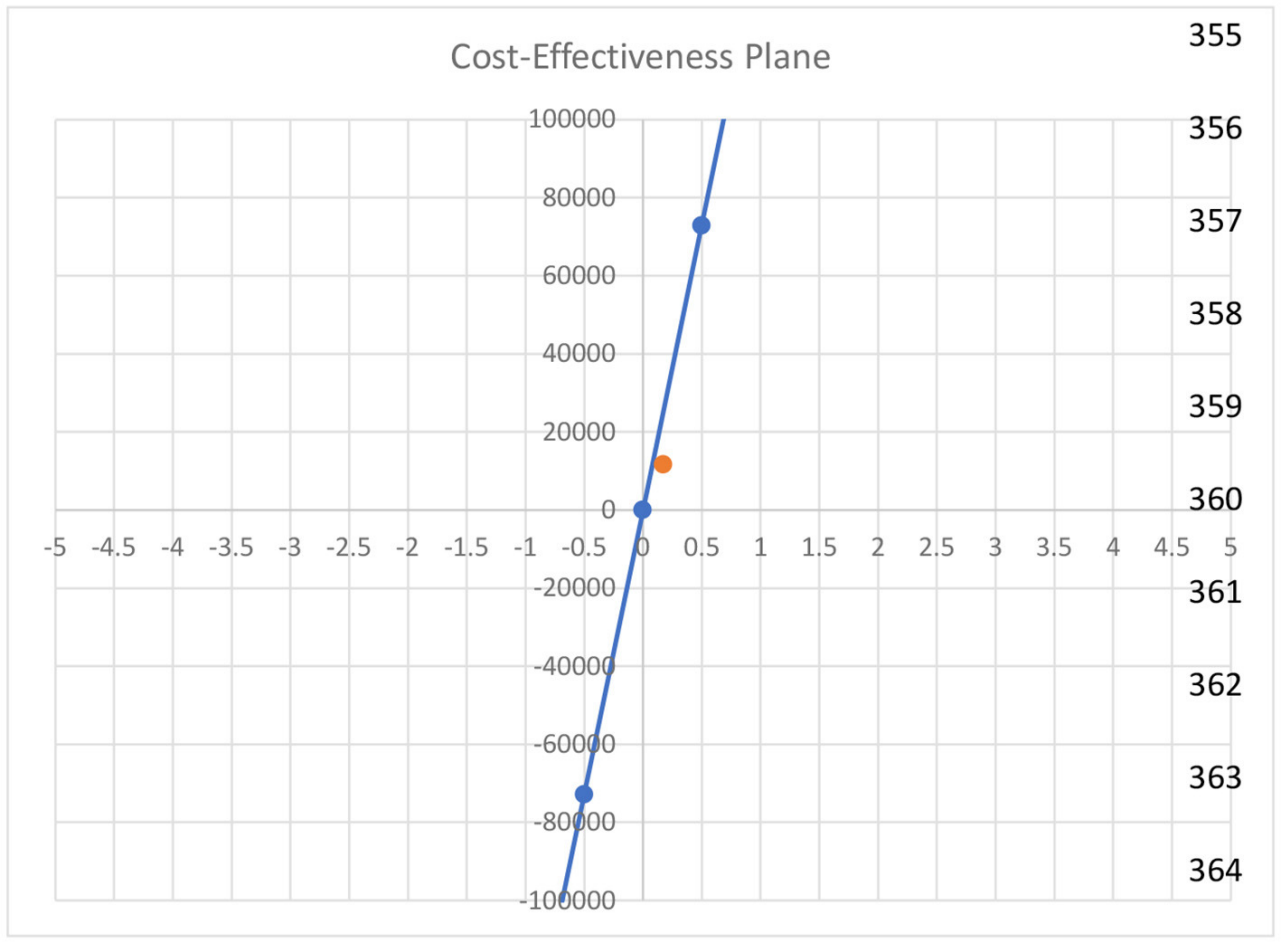
Cost-effectiveness plane of the study.

Figure 3 illustrates the cost-effectiveness plane. The orange dot indicates the ICER value that falls above the reference line and in the first quadrant. It shows that our intervention is highly cost-effective as compared to the control.

### Model input parameters

Input parameters used for the model are presented in Table 9. The cost and utility data for the intervention and the control were calculated from the primary data collection.

**Table 9:** Model input parameters.

**Parameters**	**Intervention arm**		**Control arm**		**Distribution**	**Source**
	**Base case**	**95% CI**	**Base case**	**95% CI**		
Probability of full adherence	0.896	0.878–0.914	0.422	0.414–0.43	Normal	Primary data
Probability of partial adherence	0.015	0.015–0.015	0.46	0.451–0.469	Normal	Primary data
Probability of non-adherence	0.089	0.087–0.091	0.118	0.116–0.12	Normal	Primary data
Probability of full adherence from treatment completed	0.992	0.972–1.012	0.971	0.952–0.99	Normal	Primary data
Probability of full adherence from treatment extended	0.008	0.008–0.008	0.029	0.028–0.03	Normal	Primary data
Probability of full adherence from death	0	0–0	0	0–0	Normal	Primary data
Probability of full adherence from defaulter	0	0–0	0	0–0	Normal	Primary data
Probability of partial adherence from treatment completed	1.000	0.98–1.02	0.986	0.966–1.006	Normal	Primary data
Probability of partial adherence from treatment extended	0	0–0	0.014	0.014–0.014	Normal	Primary data
Probability of partial adherence from death	0	0–0	0	0–0	Normal	Primary data
Probability of partial adherence from defaulter	0	0–0	0	0–0	Normal	Primary data
Probability of non-adherence from treatment completed	0	0–0	0	0–0	Normal	Primary data
Probability of non-adherence from treatment extended	0	0–0	0	0–0	Normal	Primary data
Probability of non-adherence from death	0.500	0.49–0.51	0.474	0.465–0.484	Normal	Primary data
Probability of non-adherence from defaulter	0.500	0.49–0.51	0.526	0.516–0.537	Normal	Primary data
Probability of QALY from full adherence	0.007	0.007–0.008	0.007	0.007–0.007	Normal	Primary data
Probability of QALY from partial adherence	0.006	0.006–0.006	0.005	0.005–0.005	Normal	Primary data
Probability of QALY from non-adherence	0.005	0.005–0.005	0.001	0.001–0.001	Normal	Primary data
Probability of overall QALY	0.006	0.006–0.006	0.007	0.007–0.007	Normal	Primary data
Cost of treatment completed	44.37	43.483–45.257	38.352	37.585–39.119	Normal	Calculated
Cost of full adherence	39.769	38.973–40.564	16.198	15.875–16.522	Normal	Calculated
Cost of full adherence_treatment completed	39.440	38.651–40.229	15.722	15.408–16.037	Normal	Calculated
Cost of full adherence_treatment extended	39.769	38.974–40.564	16.198	15.874–16.522	Normal	Calculated
Cost of full adherence_death	0	0–0	0	0–0	Normal	Calculated
Cost of full adherence_defaulter	0	0–0	0	0–0	Normal	Calculated
Cost of partial adherence	0.657	0.644–0.671	17.628	17.275–17.98	Normal	Calculated
Cost of partial adherence_treatment completed	0.657	0.644–0.671	17.390	17.042–17.737	Normal	Calculated
Cost of partial adherence_treatment extended	0	0–0	17.628	17.275–17.98	Normal	Calculated
Cost of partial adherence_death	0	0–0	0	0–0	Normal	Calculated
Cost of partial adherence_defaulter	0	0–0	0	0–0	Normal	Calculated
Cost of non-adherence	3.944	3.865–4.023	4.526	4.436–4.617	Normal	Calculated
Cost_non-adherence_treatment completed	0	0–0	0	0–0	Normal	Calculated
Cost of non-adherence_treatment extended	0	0–0	0	0–0	Normal	Calculated
Cost of non-adherence_death	1.972	1.933–2.011	2.144	2.101–2.187	Normal	Calculated
Cost of non-adherence defaulter	1.972	1.933–2.011	2.382	2.335–2.43	Normal	Calculated
Cost of per beneficiary	65.733	64.419–67.048	47.643	46.69–48.595	Normal	Calculated
Average age of participants	37	0.363–0.377	37	0.363–0.377	Normal	Primary data

### One-way sensitivity analysis (OWSA)

**Figure 4:**
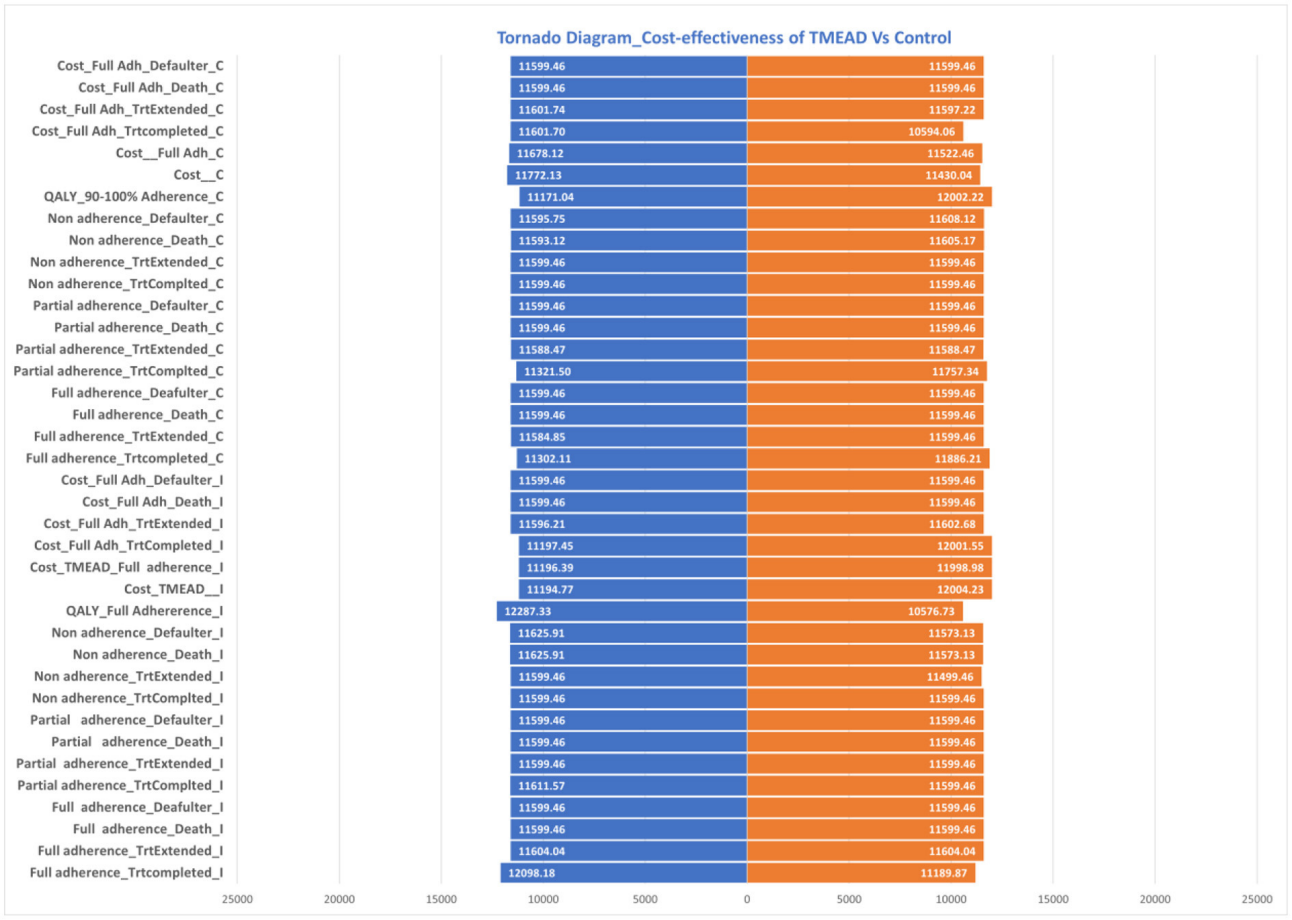
Tornado diagram of cost-effectiveness of intervention and control.

Uncertainty in the model parameter values was assessed through one-way sensitivity analysis (OWSA). OWSA was carried out in MS Excel with 95% CI values in the base case values, as shown in Table 8. The results of OSWA were presented using a tornado graph (Figure 4). The tornado diagram of one-way sensitivity analysis shows that the ICER value is slightly changed when the input parameters are changed for multiple indicators. The cost of the control arm, the cost for full adherence in the treatment completed group, QALYs among the full adherent patients in both the intervention and control arms, and the cost for defaulters among partial adherent to patients in the control arm were the key parameters that influenced the model.

## Discussion

This study assessed the adherence (clinical and digital) and cost-effectiveness of TMEAD as a tool for measuring and promoting medication adherence among DSTB patients. The participants' demographic characteristics, such as age, the ratio of men to women, and their treatment outcomes, were comparable to the pilot study done by Cross A et al. in the Mumbai region (20). This suggests that we studied a regionally representative patient sample. However, the adherence rates of our patients could have been biased by their participation in the study.

Digital health interventions are increasingly used to support TB treatment in diverse settings globally (21–23). Our study found that the use of the TMEAD device to remind patients with TB to take their drugs resulted in medication adherence of 99% compared to 90% in the standard-of-care scenario. This increase was seen for all TB treatment adherence measures in this study. The results demonstrate convincingly that the intervention strategy works, and the more the intervention received by the patients, the better the response.

Stagg et al. cited prior validation of electronic monitoring with urine rifampicin levels; the pharmacokinetics of that drug limited its interpretability (24). In line with the other digital adherence technologies, TMEAD also reports adherence, which is confirmed in this study by urine analysis. Our findings further support the adherence results as per urine analysis, for those patients' samples processed in the first cycle, 90.6% had urine rifampicin traces. At the end of the third cycle of urine sample collection in the intervention arm, 86.3% of samples were positive for urine rifampicin. While in the control arm, 77.2% of the samples were rifampicin-positive.

Even though many types of DATs exist and have been used for different disease conditions, a systematic review done by Ngwatu et al. suggests that some digital interventions can potentially improve medication adherence and patient outcomes (25). While the evidence remains incomplete and generalizability limited, the studies reviewed suggest these technologies may be at least as effective as the standard of care (26).

Cost-effectiveness analysis (CEA) has been used as a tool for addressing efficiency issues in the allocation of scarce health resources, providing as it does a method for comparing the relative costs and health gains of different (and often competing) health interventions. In our study, the incremental cost-effectiveness ratio (ICER) was INR 11,599 (USD 146) per quality-adjusted life-year (QALY), which is 0.07% of the per capita GDP of the country. Our results show TMEAD intervention is cost-effective according to the willingness-to-pay for health threshold.

## Limitations

One of the limitations of this study is the non-random selection/allocation of the study participants; hence, the study subjects were not representative of all patients taking treatment for TB in Nasik. The COVID-19 induced prolonged lockdown has resulted in migration and patient attrition. There were various implementation challenges, such as actual consumption, which could not be monitored. Problems with the mobile network resulted in difficulty in contacting patients, sending device alerts, and refilling devices.

## Conclusion

This study revealed that patient-reported treatment adherence was high in TMEAD as compared to standard therapy of care for DSTB patients and the intervention is cost-effective. This study has several important public health implications for the use of a TMEAD device in resource-limited settings. First, the evaluation of patient and health worker behaviors and beliefs following the implementation of this technology in a new setting will be essential in optimizing its acceptability and clinical impact. Second, the introduction of new technologies alone is just one part of a broader approach to adherence support. Technological innovations must be accompanied by sustainable health system strategies to address and overcome diverse barriers to treatment completion. TMEAD can complement the national strategy of TB elimination by improving adherence to the treatment regimen.

## Data availability statement

Data from this study will be available at the Indian Institute of Public Health Gandhinagar (IIPHG), India, after the completion of this study. Researchers who meet the criteria for access to confidential data are encouraged to approach SS (ssaha@iiphg.org).

## Ethics statement

The study was approved by the Technical Appraisal Committee for Health Technology Assessment of the Department of Health Research, New Delhi and the Institutional Ethics Committee of the Indian Institute of Public Health, Gandhinagar, vide TRC-IEC No: 02/2020-21 dated 29 May 2020. The patients/participants provided their written informed consent to participate in this study.

## Author contributions

SS and DS conceptualized, designed, and led the study. DS contributed in the study design and methods. DR coordinated the field data collection. NH and RD were part of the technology design team and contributed in the study. MJ, MS, and JC contributed in the study planning and execution. JS, DR, SY and HS contributed in data analysis and writing. DR, JS, and KR contributed in economic analysis and writing. All authors read, edited, and approved the final version of the manuscript.

## Footnotes

^1^Fund IH. India Health Fund. Available online at: https://www.indiahealthfund.org/.
