# Supplementary material for: Tuberculosis Monitoring Encouragement Adherence Drive (TMEAD): Toward improving the adherence of the patients with drug-sensitive tuberculosis in Nashik, Maharashtra
Source: Front Public Health. 2022 Dec 21;10:1021427. doi: 10.3389/fpubh.2022.1021427 (PMC9812554; doi:10.3389/fpubh.2022.1021427)
Supplement: Supplementary file 1 [file Table_1.DOCX]

Annexure 1

STROBE Statement—Checklist of items that should be included in reports of ***cohort studies***

|  | Item No | Recommendation | Page No |
| --- | --- | --- | --- |
| **Title and abstract** | 1 | (*a*) Indicate the study’s design with a commonly used term in the title or the abstract | Longitudinal cohort study as stated in the Abstract on page 2 and Patients and  Methods on page 4  Longitudinal cohort study as stated in the Abstract on page 2 and Patients and  Methods on page 4  Prospective cohort study as stated in the Abstract (Method) on page 2 |
|  |  | (*b*) Provide in the abstract an informative and balanced summary of what was done and what was found | Provided in Abstract on page 2. |
| Introduction | | | |
| Background/rationale | 2 | Explain the scientific background and rationale for the investigation being reported | Included in the Introduction on pages 3 and 4. |
| Objectives | 3 | State specific objectives, including any prespecified hypotheses | Included in the Introduction on page 4. |
| Methods | | | |
| Study design | 4 | Present key elements of study design early in the paper | Included in the Methods on page 5. |
| Setting | 5 | Describe the setting, locations, and relevant dates, including periods of recruitment, exposure, follow-up, and data collection | Included in the Methods on pages 4,5 and 6 |
| Participants | 6 | (*a*) Give the eligibility criteria, and the sources and methods of selection of participants. Describe methods of follow-up | Included in the Methods on pages 4, 5 and 6. |
|  |  | (*b*) For matched studies, give matching criteria and number of exposed and unexposed | Not applicable. |
| Variables | 7 | Clearly define all outcomes, exposures, predictors, potential confounders, and effect modifiers. Give diagnostic criteria, if applicable | Included in the Methods on pages 4, 5 and 6. |
| Data sources/ measurement | 8* | For each variable of interest, give sources of data and details of methods of assessment (measurement). Describe comparability of assessment methods if there is more than one group | Included in the Methods on pages 5 and 6. |
| Bias | 9 | Describe any efforts to address potential sources of bias | Addressed in the limitations paragraph on page 16 |
| Study size | 10 | Explain how the study size was arrived at | Included in the Methods on page 5 (sample was derived from a fixed geographical catchment area) |
| Quantitative variables | 11 | Explain how quantitative variables were handled in the analyses. If applicable, describe which groupings were chosen and why | Included in the Methods on pages 4, 5 and 6 |
| Statistical methods | 12 | (*a*) Describe all statistical methods, including those used to control for confounding | Included in the Methods on pages 4, 5 and 6 |
|  |  | (*b*) Describe any methods used to examine subgroups and interactions | Included in the Methods on pages 4, 5 and 6 |
|  |  | (*c*) Explain how missing data were addressed | Included in the Methods on pages 4, 5 and 6 |
|  |  | (*d*) If applicable, explain how loss to follow-up was addressed | Included in the Methods on pages 4, 5 and 6 |
|  |  | (*e*) Describe any sensitivity analyses | Included in the Methods on page 8 |
| Results | | |  |
| Participants | 13* | (a) Report numbers of individuals at each stage of study—eg numbers potentially eligible, examined for eligibility, confirmed eligible, included in the study, completing follow-up, and analysed | Included in the Results on page 12 |
|  |  | (b) Give reasons for non-participation at each stage | Not applicable. |
|  |  | (c) Consider use of a flow diagram | Not required. |
| Descriptive data | 14* | (a) Give characteristics of study participants (eg demographic, clinical, social) and information on exposures and potential confounders | Included in the Results on pages 9 and 10. |
|  |  | (b) Indicate number of participants with missing data for each variable of interest | Included in the Results on pages 9,10 and 11. |
|  |  | (c) Summarise follow-up time (eg, average and total amount) | Included in the Results on page 12. |
| Outcome data | 15* | Report numbers of outcome events or summary measures over time | Included in the Results on pages 12,13 14 and 15, and summarized in Tables 3,4,5 and 6. |

| Main results | 16 | (*a*) Give unadjusted estimates and, if applicable, confounder-adjusted estimates and their precision (eg, 95% confidence interval). Make clear which confounders were adjusted for and why they were included | Not applicable |
| --- | --- | --- | --- |
|  |  | (*b*) Report category boundaries when continuous variables were categorized | Not applicable |
|  |  | (*c*) If relevant, consider translating estimates of relative risk into absolute risk for a meaningful time period | Not applicable. |
| Other analyses | 17 | Report other analyses done—eg analyses of subgroups and interactions, and sensitivity analyses | Not applicable. |
| Discussion | | | |
| Key results | 18 | Summarise key results with reference to study objectives | Included in the Discussion on page 20. |
| Limitations | 19 | Discuss limitations of the study, taking into account sources of potential bias or imprecision. Discuss both direction and magnitude of any potential bias | Included in the Discussion on pages 21. |
| Interpretation | 20 | Give a cautious overall interpretation of results considering objectives, limitations, multiplicity of analyses, results from similar studies, and other relevant evidence | Included in the Discussion on pages 20 and 21. |
| Generalisability | 21 | Discuss the generalisability (external validity) of the study results | Included in the Discussion on page 21. |
| Other information | | | |
| Funding | 22 | Give the source of funding and the role of the funders for the present study and, if applicable, for the original study on which the present article is based | Provided in the text on page 22. |

*Give information separately for exposed and unexposed groups.

**Note:** An Explanation and Elaboration article discusses each checklist item and gives methodological background and published examples of transparent reporting. The STROBE checklist is best used in conjunction with this article (freely available on the Web sites of PLoS Medicine at http://www.plosmedicine.org/, Annals of Internal Medicine at http://www.annals.org/, and Epidemiology at http://www.epidem.com/). Information on the STROBE Initiative is available at http://www.strobe-statement.org.
